# Endogenous Matrix-Derived Inhibitors of Angiogenesis

**DOI:** 10.3390/ph3103021

**Published:** 2010-09-28

**Authors:** Malin Sund, Pia Nyberg, Hans Petter Eikesdal

**Affiliations:** 1 Department of Surgery, Umea University Hospital, Umea University, SE-90185 Umea, Sweden;; 2 Department of Diagnostics and Oral Medicine, Institute of Dentistry, University of Oulu, Oulu, Finland; E-Mail: pia.nyberg@oulu.fi (P.N.); 3 Department of Oncology, Institute of Medicine, Haukeland University Hospital, University of Bergen, Bergen, Norway; E-Mail: hans.eikesdal@biomed.uib.no (H.P.E.)

**Keywords:** angiogenesis, extracellular matrix, collagen, cancer, therapy, biomarker

## Abstract

Endogenous inhibitors of angiogenesis are proteins or fragments of proteins that are formed in the body, which can inhibit the angiogenic process. These molecules can be found both in the circulation and sequestered in the extracellular matrix (ECM) surrounding cells. Many matrix-derived inhibitors of angiogenesis, such as endostatin, tumstatin, canstatin and arresten, are bioactive fragments of larger ECM molecules. These substances become released upon proteolysis of the ECM and the vascular basement membrane (VBM) by enzymes of the tumor microenvironment. Although the role of matrix-derived angiogenesis inhibitors is well studied in animal models of cancer, their role in human cancers is less established. In this review we discuss the current knowledge about these molecules and their potential use as cancer therapeutics and biomarkers.

## 1. Introduction

In the last decade the importance of the tumor stroma in cancer progression and metastasis has become increasingly clear. The stroma is defined as the non-malignant cellular component of a cancer as well as the extracellular matrix (ECM) of the tumor [1,2]. The ECM in a tumor can be produced and modified by both stromal and cancer cells. For many solid tumors the stromal compartment actually makes up the bulk of the tumor volume and is of importance for the development of a cancer from a malignantly transformed cell [3]. The stromal cells of a tumor are fibroblasts, cells of the immune system and cells of the tumor vasculature and tumor lymphatic vessels, namely the endothelial cells (ECs), pericytes and lymphatic endothelial cells (LECs) [1,4]. 

**Figure 1:**
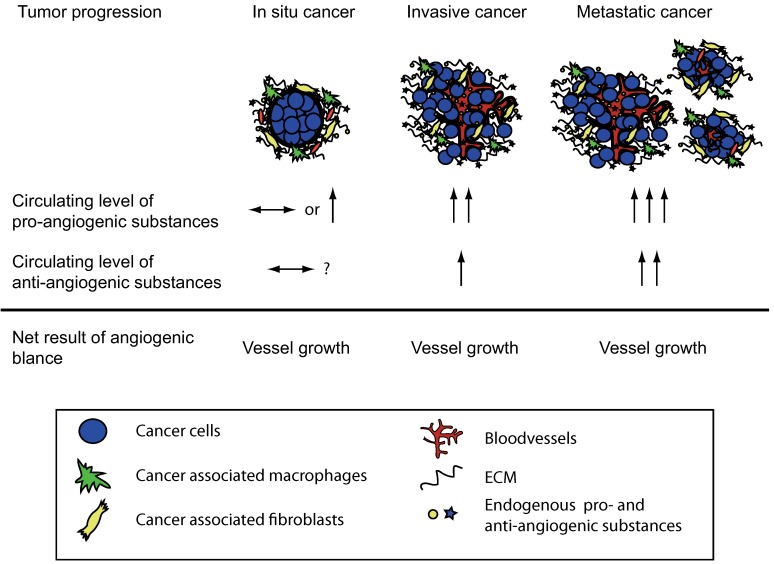
The angiogenic balance and the effect of tumor load. During tumor progression the total concentration of pro-angiogenic molecules is higher than that of anti-angiogenic molecules. This allows for new vessel development and further tumor growth. Note that the total level of the anti-angiogenic factors can be higher in a cancer patient when compared to a healthy individual. However, the pro-angiogenic stimuli are even higher and therefore the increased level of the anti-angiogenic molecules is a reflection of the tumor load.

A central stromal function is the development of a tumor vasculature through the process of angiogenesis, in which new blood vessels are formed from previous vessels. Angiogenesis has been reviewed extensively elsewhere [5,6,7,8,9,10], and is influenced by a multitude of angiogenesis stimulators such as vascular endothelial growth factor (VEGF), and inhibitors that together make up the angiogenic balance [7,9]. Endogenous matrix-derived angiogenesis inhibitors are bioactive protein fragments cleaved from larger ECM proteins, such as type IV and XVIII collagens. These protein fragments are generated during the remodeling of the ECM in the tumor microenvironment and/or during angiogenesis [7,9,11]. Many of these molecules are found in the circulation of healthy individuals. It is believed that these substances are important physiologic angiogenesis inhibitors but that during tumor growth the angiogenic balance is tilted towards pro-angiogenesis through an overproduction of pro-angiogenic factors [7,9,11]. It has also been shown that in many cancers the matrix-derived anti-angiogenic substances increase and reflect the tumor load [7,12]. Although the level of anti-angiogenic factors increase, it is still believed that the simultaneous overproduction of the pro-angiogenic substances is higher thus allowing for continued angiogenesis and subsequent tumor growth (Figure 1). Therefore, although the protelytic activity of various proteases, such as MMPs and caspases, is a key feature of malignancy and invasiveness, some of these proteases also are involved in the release of matrix-derived anti-angiogenic molecules. Subsequently suppressing the activity of these proteases can lead to enhanced angiogenesis and increased tumor growth. This dual role of proteases in the tumor microenvironment has been shown in many studies [13,14,15,16,17] .

As angiogenesis clearly is very central for the conversion of a dormant *in situ* tumor to an aggressive cancer with the capability to metastasis, there has been a great interest to develop therapies interfering with this process and thereby inhibiting tumor growth. One can in theory prevent angiogenesis by interfering with (and thus reducing the effect of) pro-angiogenic molecules, or by increasing the level of anti-angiogenic molecules [18,19,20]. So far all anti-angiogenic therapeutic agents in the clinic are of the former sort. Although many anti-angiogenic substances, such as VEGF neutralizing antibodies and tyrosine kinase inhibitors, have entered the clinic in recent years, unfortunately, the effect of these substances has in general been quite modest [21,22,23]. This has been attributed to the fact that these drugs so far have been used in patients with advanced cancer and it is well known from preclinical studies that a tumor at this stage is not so responsive to treatment with a single anti-angiogenic agent [23]. However, currently there are many on-going trials with these substances in an adjuvant setting for operable cancers with the idea of preventing the further progression of micrometastases. Other problems encountered have been the observed rebound effects after treatment as well as the problems with selecting patients for these agents due to the lack of good predictive biomarkers for anti-angiogenic therapy [22,24].

Currently there are no validated biomarkers that predict response to antiangiogenic agents clinically [23,24]. Such biomarkers are clearly needed as this group of drugs rarely induces tumor regression, and conventional radiologic evaluation markers, such as partial and complete response are therefore less applicable. There is currently a focus on measuring endothelial growth factors (such as VEGF) and circulating endothelial cells in the blood as surrogate markers for response [24]. When patients with glioblastoma were given a pan-VEGF receptor tyrosine kinase inhibitor, changes in collagen IV in the blood correlated with progression free survival and overall survival [25]. This points to a potential for measuring collagen IV degradation products in the clinic for the use as prognostic markers or to predict response to antiangiogenic therapy. 

In this review we will discuss the matrix-derived anti-angiogenic inhibitors cleaved from the ECM proteins type IV and XVIII collagen. Type IV collagen is the main protein component of all basement membranes (BMs) and is crucial for the stability and assembly of this specialized ECM [11,26,27]. Type IV collagen is composed of six different type IV collagen α-chains in mammals, the α1- and α2-chains are found in most basement membranes, whereas the other chains display more restricted expression patterns in various tissues. Type XVIII collagen is a heparan sulphate proteoglycan (HSPG) found in most vascular and other basement membranes [11,28]. We will review the current knowledge of how these molecules exert their effect on the endothelial cells, how they affect tumor growth in animal models, and what is known about the role of these substances in human cancer progression. Additionally, we will discuss their potential use as tumor biomarkers or as cancer therapeutics in human cancer.

## 2. Type XVIII Collagen Derived Endostatin

### 2.1. Cellular source and effects—in vitro and in vivo studies

Endostatin is a 20 kDa C-terminal fragment of type XVIII collagen with potent anti-angiogenic activity, as shown by both *in vitro* and *in vivo* studies. Type XVIII collagen is a protein found in most basement membranes (BMs) in the body, including the vascular basement membrane (VBM) [29]. Endostatin can be cleaved from type XVIII collagen by several proteases found in the tumor microenvironment, such as MMPs, cathepsin-L, elastase [30,31,32]. Increased circulating endostatin is found in many forms of cancer, but can also be detected physiologically in healthy individuals [28,33]. The physiological levels of endostatin vary depending on whether plasma or serum is used, and this has been attributed to due to the shown scavenging of endostatin by platelets [34,35].

The NC1-domain of type XVIII collagen consists of an N-terminal association domain of about 60 residues, followed by a triple helical domain and at the C-terminal area the 180-residue endostatin domain. A flexible hinge region containing several protease-sensitive segments connects the C- and N-terminus, and cleavage at this site will lead to release of endostatin from type XVIII collagen [36]. High-resolution X-ray structures for endostatin [37,38] show a structure with a globular fold of 3-nm diameter containing 16 *β* strands, two *α* helices and two disulfide bridges. A single zinc ion is bound to the *N*-terminal region of endostatin and involves five residues, H132, H134, H142, D136, and D207 [36,38]. The binding generates three variant confirmations of the *N*-terminus, which indicates a structural role for the zinc. The importance of increased endostatin stability is highlighted by the apparently better effect of a N-terminally modified variant of endostatin (discussed later in this review) as a cancer therapeutic, in which an additional zinc-binding domain has been added to the sequence. 

A surface patch of 11 arginines on endostatin explains its heparin binding capacity. This heparin-binding site has been mapped to six arginine residues of endostatin [39], and been found to take place at two sites, a primary (R155, R158, R184, R270) and a secondary binding site (R193 and/or R194). However, an efficient binding of endostatin to heparin requires a simultaneous binding to both sites [39]. Endostatin has been shown to bind to many BM proteins, such as laminin, nidogen, fibulin and perlecan in both solid phase and surface plasmon resonance assays [40,41]. 

Numerous studies have been conducted in order to understand how endostatin exerts its effect on tumor vasculature. From these studies it is clear that endostatin affects ECs in many different ways. Released endostatin binds to its receptors on the endothelial cells such as α5β1, αvβ3, αvβ5 integrins and glypicans [42,43,44]. This binding leads to an inhibition of endothelial cell proliferation, migration and induction of apoptosis [45,46,47,48]. Endostatin binding to α5 integrin causes an inhibition of the focal adhesion kinase (FAK)/c-Raf/MEK1/2/p38/ERK1 mitogen-activated pathway [44]. Additionally the endostatin-α5 integrin binding leads to down-regulation of RhoA and subsequent disruption of focal adhesions and actin stress fibers [49,50]. Endostatin has been shown to interfere with the binding of VEGF to VEGFR2 and subsequently the tyrosine phosphorylation of the receptor [51]. By inhibiting MMP-2 in the ECM, endostatin also directly affects matrix remodeling [52]. 

Mice deficient of type XVIII collagen and thus also of the endostatin domain appear surprisingly quite normal, and do not show signs of major vascular malformations or spontaneous tumor growth [53]. However, these mice do exhibit vascular developmental defects in the eye highly similar to those observed in the human disease Knobloch syndrome, which is caused by mutations in type XVIII collagen [54]. Additionally, aortic ring explants from these mice display increased microvessel outgrowth, indicating a shift in the angiogenic balance in the mice deficient of endostatin [55]. This shift in the angiogenic balance is further emphasized by the finding that endostatin deficient mice implanted with type XVIII collagen negative cancer cells display increased tumor growth [56]. Additionally, overexpression of circulating endostatin in transgenic mice leads to reduced tumor growth and vascularization [56]. Also, the effect of endostatin is not limited to the endothelial cells, as carcinogen-induced skin tumors in mice over-expressing endostatin in the skin lead to a significant reduction in lymphatic vessels and an inhibition of lymph node metastasis besides the expected inhibition of tumor angiogenesis in early stage tumors [57]. 

Endostatin has been shown to be very effective in treating mouse tumors using various tumor models [46,58]. In these studies both recombinant human and mouse endostatin has been used which indicates that the effects on the tumor vasculature are quite well conserved between species. The studies in tumor bearing mice have shown that the treatment is most effective at an early stage of tumor development and when the treatment is given as a continuous infusion as compared to bolus injections [58]. The concentration of recombinant endostatin has ranged from 10-100 mg/kg/day and without any signs of toxicity even during long treatment. These findings naturally led to an interest of trying endostatin as a cancer therapeutic in human cancer.

### 2.2. Role in human cancer diagnostics and therapy

The expression pattern and circulating levels of endostatin have been studied for many human cancers. For most cancers high expression of endostatin by the tumor as well as high circulating levels indicates poor prognosis, likely reflecting a large tumor burden [7,12,59,60,61,62,63,64]. Therefore, although the level of the anti-angiogenic endostatin is higher than in the healthy control, the total level of pro-angiogenic substances are even higher, which allows for sustained angiogenesis and further tumor growth (Figure 1). Nevertheless, most of these studies clearly indicate that analysis of endostatin levels can be of value in the analysis of disease progression and prognosis. We have shown that the levels of endostatin in patients with pancreatic cancer are increased at the time of diagnosis and decrease after treatment with surgery or chemotherapy [12]. 

Three phase I clinical trials have been published in which recombinant human endostatin was used to treat patients with many types of metastatic cancer [33,65,66]. In these trials endostatin was given as a daily intravenous infusion with concentrations ranging from 15 to 600 mg/m^2^. The effect on tumor growth was much lower than what was expected based on the previous experimental studies in mice [46,58,67,68,69]. The reasons for lack of effect are believed to be the trial design. The included patients had very advanced tumors [33,67], the mode of administration was inadequate [46,58], and there was a need for a modification of the molecules in order to increase activity/stability [70]. A phase II trial for endostatin treatment of pancreatic neuroendocrine tumors was subsequently done based on the findings from the phase I studies. However, treatment with endostatin did not result in significant tumor regression in patients with advanced neuroendocrine tumors [71]. These mostly negative trials naturally led to a pessimism regarding the possibility to use endostatin as a cancer therapeutic. Most interestingly however endostatin has re-entered the clinic with a modification to the original sequence by the addition of an additional zinc-binding site at the N-terminus. This modification results in a more stable molecule with similar anti-angiogenic activity [70]. This modified endostatin is now used in certain countries for the treatment of lung and gastric cancer [72,73]. Interestingly, it has recently been shown that the levels of circulating endothelial cells and survivinin may be ideal markers predicting efficacy of endostatin treatment in patients with lung cancer [74]. There are currently 15 clinical trials listed for the modified endostatin on www.clinicaltrials.gov in combination with other treatments, and used both in a neo-adjuvant setting as well as in treatment of advanced cancers. Although a couple of these studies are already completed, most are still recruiting and no published results have yet been presented.

## 3. Type IV Collagen Derived Anti-Angiogenic Substances

### 3.1. Tumstatin

Tumstatin is an antiangiogenic endogenous 28kDa protein fragment derived from the non-collagenous (NC1) domain of the α3-chain of type IV collagen (collagen IV-α3) [75]. The α3-chain of type IV collagen has a more restricted expression pattern than the α1- and α2-chains, and is mainly found in the specialized basement membranes of the kidney, lung and testis [76,77]. Tumstatin inhibits endothelial cell proliferation through direct inhibition of protein synthesis causing endothelial cell apoptosis [75,78]. The antiangiogenic activity of tumstatin is restricted to amino acids 74–98; named the T7 or tumstatin peptide and inhibition of protein synthesis in endothelial cells is mediated by this tumstatin peptide moiety [78,79,80]. Accordingly, the tumstatin peptide and the full tumstatin protein have equivalent anti-proliferative effects on endothelial cells [81]. Tumstatin binds to αvβ3 integrin on ECs via the tumstatin peptide moiety, and this binding is pivotal for its anti-angiogenic activity [80,82]. Using the known crystal structure of αvβ3 integrin and the NC1 domain hexamer of collagen IV, a potential interaction interface between the tumstatin peptide and a groove on the β3 subunit of αvβ3 integrin has been shown by 3D homology modeling [80]. One of the proteases involved in cleavage of tumstatin from the α3-chain is MMP-9. It has been shown that mice deficient of MMP-9 have reduced levels of circulating tumstatin, which subsequently lead to increased pathological angiogenesis and tumor growth when these mice were implanted with tumors (Hamano *et al*.). However, the exact cleavage site and whether other MMPs and proteases are involved remain unknown.

Whereas the NC1 domain of the α3 chain of type IV collagen, wherein tumstatin resides, potently inhibits EC proliferation, the NC1 domain of the α5 chain of collagen IV lacks antiangiogenic activity [83]. Through sequence comparison of the NC1 domains of the α3 and α5 chains of collagen IV we sought to identify the essential amino acids necessary for tumstatin endothelial cytotoxicity. By substituting the amino acids LVD to MIN[L→M (66); V→I (70); D→N (72)] in the tumstatin peptide, the tumstatin peptide mutant was generated and this mutant no longer inhibited EC protein synthesis [78]. Recently, the anti-angiogenic and anti-tumor activity of tumstatin peptide and the tumstatin peptide mutant were compared [80]. Whereas the mutant peptide could still bind to ECs, its antiangiogenic and anti-tumor activity was lost. In a much wider assessment we systematically synthesized a series of mutant tumstatin peptides to mimic the α5-chain amino acid sequence, and thereby identified amino acids crucial for tumstatin activity [80]. 

Apart from endothelial cell cytotoxicity, tumstatin also inhibits the growth of several tumor cell lines *in vitro*. While the EC cytotoxicity of tumstatin resides in the N-terminal half of tumstatin, the C-terminal half exhibits direct tumor cell cytotoxicity [81,82]. The NC1 domain of the α3-chain of type IV collagen, in which the tumstatin protein resides, was found to inhibit the growth of human melanoma cells *in vitro* [84]. In a recent study, the T3 peptide of tumstatin, which contains a part of the T7 peptide, was shown to inhibit glioma cell growth *in vitro*, if the tumor cells did not have a mutated PTEN/high expression of phosphorylated Akt [85]. 

*In vivo* data demonstrates that both tumstatin and tumstatin peptide exhibit anti-angiogenic and anti-tumor activity in mice. Tumstatin was shown to inhibit the growth of both 786 human renal cell carcinoma and PC3 prostate cancer in nude mice [75]. Recombinant tumstatin, produced in *E.coli*, inhibited angiogenesis *in vitro* and *in vivo*, and suppressed the growth of orthotopically implanted B16F10 melanomas and oral squamous cell carcinomas [86,87]. Using orthotopic injection of MDA MB435 breast carcinoma, the antiangiogenic tum5 domain of tumstatin, in which the T7 peptide resides, also demonstrated anti-tumor activity [81]. Furthermore, the addition of an anti-VEGF antibody to tumstatin peptide therapy augments its effect and is a potential way of circumventing acquired resistance to such angiogenesis inhibitors [80,88]. 

Implementing tumstatin clinically also requires sufficient bioavailability. Tumstatin itself has low solubility, but the conjugation of tumstatin or tumstatin peptide to various solubilizing proteins increases the solubility, while maintaining its activity [80,89]. Also, the design of shorter peptide fragments of proteins like tumstatin, with preserved biological activity, will facilitate large scale production of these compounds for later clinical use [90]. 

Clinical and preclinical data clearly demonstrates that antiangiogenic therapy against malignant tumors eventually fails, due to various resistance mechanisms [88]. The need for additional angiogenesis inhibitors is therefore obvious, and combinations of compounds targeting different endothelial cell growth mechanisms is one potential way of counteracting drug resistance [88]. We combined the tumstatin peptide with an anti-VEGF antibody and demonstrated an extensive improvement in anti-tumor efficacy, compared to tumstatin peptide alone [80]. 

There is limited knowledge of how mutations in genes of the endogenous angiogenesis inhibitors would affect cancer progression. Mutations in the α3-chain of type IV collagen is well known from Alport disease, an inheritable disease wherein patients suffer from progressive renal failure, sensorineural deafness and ocular problems [91,92], but there is no link thus far between such type IV collagen mutations and the risk of cancer. Also, mutations in the tumstatin moiety of the α3-chain have not been reported, but one could speculate that inactivating tumstatin mutations, like those generated artificially in the lab [80], would remove an important brake on the angiogenesis process and facilitate tumor growth. A range of different single nucleotide polymorphisms (SNPs) in the α3-chain of collagen IV are also known from healthy individuals, but are of unknown significance as of today [91]. 

### 3.2. Arresten

Arresten is a 26 kDa anti-angiogenic non-collagenous fragment derived from the α1-chain of type IV collagen. Together with the α2-chain, the α1-chain is the most ubiquitously distributed type IV collagen chain. It is expressed in nearly all BMs, including vascular, bronchial, alveolar and glandular basement membranes [76,77]. Arresten inhibits proliferation, migration, and tube formation of many types of endothelial cells (C-PAE, HUVEC and mouse microvascular lung endothelial cells) [93,94,95,96]. Arresten significantly increases apoptosis of microvascular endothelial cells by regulating mitochondrial signaling molecules of the Bcl-family. The pro-apoptotic effect is mediated by decreasing the expression of anti-apoptotic signaling molecules Bcl-2 and Bcl-xL [96]. The inhibition of Bcl-2 and Bcl-xL expression as well as activation of caspase-3/poly (ADP-ribose) polymerase via negatively impacting FAK/p38-MAPK signaling has recently been demonstrated also in retinal ECs [97]. However, whether arresten is in soluble or immobilized form seems to be very critical, as immobilized arresten had no effect on endothelial cell adhesion or migration [83]. 

Arresten binds to α1β1 integrin and heparan sulphate proteoglycans (HSPG) on the endothelial surface [93]. We have later shown that α1β1 integrin is a functional receptor of arresten on HUVEC endothelial cells and microvasculature essential for tumor blood supply [95,96]. The binding of arresten to α11 integrin inhibits phosphorylation of FAK. This leads to inhibition of Raf/MEK/ERK1/2/p38 MAPK pathways followed by inhibition of hypoxia inducible factor (HIF-1α) and VEGF expression, resulting in inhibition of endothelial cell migration, proliferation, and tube formation [95]. Integrin α1 is also required for the anti-survival effect of arresten [96]. In addition to the high affinity binding to α1β1 integrin, arresten also binds to HSPG on the endothelial cells, but it is not yet known how significant this binding is [93]. It is thus possible that arresten has several receptors, or receptor binding sites and affects many distinct cell-signaling pathways that together contribute to the anti-angiogenic activity. 

The endothelial cell recognition sequence of arresten seems to reside within the last 113 amino acids in the C-terminus, since it was shown to be more potent in the anti-proliferative and pro-apoptotic efficacy [96], but that does not exclude that other parts of arresten could participate in the inhibition of angiogenesis or tumor growth in distinct pathways. The three-dimensional structure of α1(IV)NC1 domain has been elucidated [98,99,100], but it is not fully known how different the conformation is in the non-helical form [101] or what the effects of binding to integrin α1β1 or other possible receptors are on arresten structure. 

*In vivo* arresten inhibits Matrigel neovascularization in mice [96]. Furthermore, it inhibits the growth of human tumors in nude mice as well as xenograft tumors in various mouse strains [93,95,96]. Arresten treatment also reduces the number of pulmonary metastatic nodules after cancer cell injections through the tail vein of mice [93]. However, when hamster melanoma cells are applied on chicken chorioallantoic membranes (CAMs) of chicken embryos, treatment with arresten does not inhibit tumor growth [83]. In mice, the importance of α1β1 integrin was confirmed by *in vivo* tumor burden studies; the tumors implanted on integrin α1 deficient mice show no integrin α1 positive vasculature, and consequently the growth of tumors and blood vessels in these mice is not inhibited by arresten regardless of what mouse strain or tumor cell line is used [95,96]. Integrin α1β1 is considered to be particularly important in pathological angiogenesis, since blocking its function by antibodies selectively inhibits VEGF-driven angiogenesis *in vivo* without any effects on the pre-existing vasculature [102]. Interestingly, arresten binding to integrin α1β1 inhibits HIF-1α synthesis and thus leads to inhibition of VEGF expression [95]. Although the pro-apoptotic effect of arresten seems to be endothelial cell specific *in vitro*, in a xenograft mouse tumor burden model arresten induces apoptosis both in endothelial cells and in tumor cells [96]. This might be an indirect effect induced by the lack of blood vessels promoting tumor cell apoptosis or alternatively arresten has a direct pro-apoptotic effect on some tumor cells *in vivo*. It is also possible that arresten is differentially processed *in vivo* and *in vitro* resulting in exposure of novel cryptic receptor binding sites. As arresten seems to be an even more potent inhibitor of angiogenesis than endostatin [93] and additionally might exhibit cytotoxicity towards the tumor cells, the protein fragment has a good potential for therapeutic use.

### 3.3. Canstatin

Canstatin was identified as a fragment of the NC1 domain of the α2-chain of type IV collagen [103]. Canstatin inhibits endothelial cell (EC) proliferation, migration and tube formation *in vitro*, and induces EC apoptosis [103,104]. Based on sequence homology between the C- and N-terminal parts of tumstatin and canstatin, it was speculated that the antiangiogenic activity of canstatin is restricted to the N-terminal part of the molecule, like in tumstatin [104]. However, *in vitro* results demonstrate that the C-terminal part of canstatin inhibits endothelial proliferation, whereas the N-terminal part seems responsible for the potent induction of endothelial apoptosis [104,105]. 

Apoptosis induced by canstatin is inflicted through the inhibition of Akt, focal adhesion kinase (FAK) and mammalian target of rapamycin (mTOR) signaling, causing upregulation of procaspase 8 and 9 and downregulation of the anti-apoptotic protein FLIP [103,106]. Furthermore, canstatin induces Fas ligand (FasL) expression and Fas-dependent apoptosis [106]. Whereas endothelial cells are targeted by canstatin, the initial publications indicated no apparent cytotoxicity against non-endothelial cells [103,104,107]. However, in a later publication it was found that canstatin induces apoptosis both in endothelial and tumor cells, by cleavage of mitochondrial procaspase-9 [108]. This procaspase-9 activation was elicited through crosstalk between the αvβ3 and αvβ5 integrin receptors, pointing to the potential functional receptors for canstatin [108]. 

The degradation of the endogenous angiogenesis inhibitors canstatin and arresten seems related to the cysteine protease cathepsin S [109]. Cathepsin S was found to degrade canstatin and arresten *in vitro*, and spontaneous RIP1-Tag2 tumors grew faster in mice that had normal levels of cathepsin S. In cathepsin S deficient mice increased protein levels of canstatin and arresten, but not tumstatin, was found in the tumor tissue, and the tumor growth was inhibited [109]. However, in a recent publication using both a mouse model for prostate cancer (TRAMP) as well as human prostate cancer material no correlation was found between increased cathepsin S levels and the expression of matrix-derived fragments of type IV collagen [110].

Canstatin inhibits tumor progression in different human xenografts, through a reduction in microvessel density, demonstrating its antiangiogenic mode of action [103,111,112]. It also delays the metastatic process, as observed in mice with B16F10 melanomas [113]. Both the N-terminal and C-terminal part of canstatin exhibits anti-tumor activity in B16 murine melanomas [104,105], but the reason for tumor response *in vivo* was not assessed in these experiments. Furthermore, canstatin augments the anti-tumor activity of ^131^I radiation therapy [112]. Whereas radiation alone upregulates HIF1 signaling to promote radioresistance, the addition of canstatin interacts with HIF1 signaling and causes apoptosis [112]. In another study the upregulation of integrin αvβ3 and αvβ5 caused by radiotherapy sensitizes various malignant tumors in mice to canstatin, facilitating disorganization of the tumor vasculature and extensive tumor cell apoptosis [113]. Canstatin has also been shown to increase the anti-tumor effect of tumor necrosis factor-related apoptosis-inducing ligand (TRAIL) gene therapy [114].

The canstatin protein can be recombinantly produced in *E. coli* or *Drosophila melanogaster* S2 cells, allowing the *ex vivo* production of the protein for *in vivo* use [103,104,105,115]. The canstatin protein can also be produced by adenovirus by injection of virus into the tumor tissue [112]. However, there still is a shortage of *in vivo* data on canstatin and how it might affect cancer progression.

### 3.4. Tetrastatin, pentastatin and hexastatin

The remaining type IV collagen chains, namely α4-, α5- and α6-, are more limited in distribution than the α1- and α2-chains, but all of them have been reported to be associated with vascular basement membranes [76,77]. The NC1 domains of these chains as well as smaller peptides derived from them have been screened for anti-angiogenic activity [83,116,117]. The full NC1 domains of α4- and α5-chains seem to lack anti-angiogenic activity when endothelial cell (HUVEC) adhesion and migration on immobilized α4 and α5 NC1 domains have been assessed. Furthermore, they do not inhibit bFGF induced angiogenesis or tumor growth in the chicken chorioallantoic membrane (CAM) assays [83]. However, short peptides derived from both the α4(NC1) and α5(NC1) domains, named tetrastatin-1-3 and pentastatin-1-3, possess anti-angiogenic activity. The pentastatin peptides efficiently inhibit HUVEC proliferation, and VEGF-induced migration. The tetrastatin peptides also potently inhibit endothelial cell migration, but have only a mild effect on proliferation [117]. The NC1 domain of the α6-chain is also called hexastatin and has been found to regulate EC adhesion, migration and proliferation. Immobilized hexastatin promotes HUVEC adhesion and migration [83], but soluble hexastatin significantly and dose-dependently inhibits endothelial cell proliferation [116]. Endothelial cell adhesion to hexastatin is almost completely blocked by anti-integrin αvβ3 antibody. Hexastatin amino acid sequence does not contain the RGD recognition motif for integrin αvβ3, suggesting that the binding is RDG-independent [83]. Small hexastatin derived peptides almost completely block HUVEC migration, but have little if any effect on proliferation [117]. Angiogenesis is significantly inhibited by hexastatin both in CAM angiogenesis model and Matrigel plug assay. CAM tumor assays as well as several mouse tumor burden models (subcutaneous transplantation of Lewis Lung carcinomas in syngeneic mice and spontaneous pancreatic insulomas developed in the RipTag2 mice) also demonstrate the inhibition of tumor growth and microvascular density by hexastatin [83,116]. 

## 4. Conclusions

Endogenous matrix-derived inhibitors of angiogenesis are molecules that are naturally produced and circulate in the body. These molecules are important in maintaining the angiogenic balance, which influences the rate of blood vessel formation. The vascular basement membrane (VBM) is a specialized extracellular matrix that surrounds all blood vessels and consists of many structural proteins such as collagens. Type IV and XVIII collagen are found in most VBMs and BMs. These proteins are degraded during angiogenesis and remodeling of the tumor stroma, which leads to he release of protein fragments with anti-angiogenic activity. Endostatin is a fragment of type XVIII collagen and tumstatin, canstatin, arresten, hexastatin, tetrastatin and pentastatin fragments of type IV collagen. These molecules are attractive candidates for potential cancer therapy due to low toxicity and promising efficacy results in animal studies. Endostatin in a modified, more stable form is currently being tested as a cancer therapeutic in multiple clinical trials. Besides their potential use as cancer therapeutics the matrix-derived angiogenic substances could function, and should be further studied as biomarkers for the efficacy of anti-angiogenic therapy. In this setting they could potentially aid in patient selection and follow-up for these therapies.

**Figure 2:**
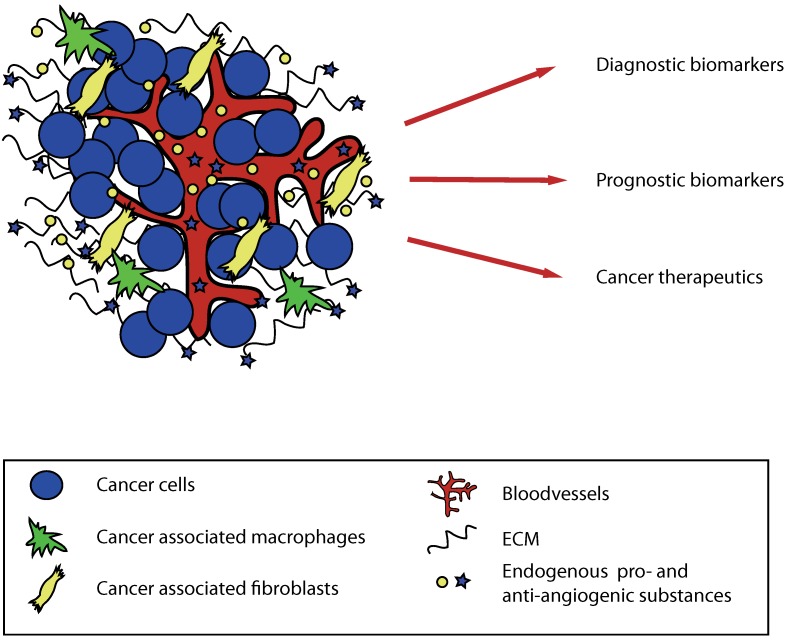
The potential use of matrix-derived anti-angiogenic molecules in human cancer. Substances cleaved from extracellular matrix proteins during tumor progression can be used as tumor markers and might also function as diagnostic and prognostic factors. These substances can also be given as therapeutics.
